# Modelling memory decay after injuries using household survey data from Khartoum State, Sudan

**DOI:** 10.1186/s12874-018-0523-9

**Published:** 2018-06-20

**Authors:** Ivar Heuch, Safa Abdalla, Sally El Tayeb

**Affiliations:** 1 Department of Mathematics, University of Bergen, P.O. Box 7803, 5020 Bergen, Norway; 2 Independent Scholar, 13 Allendale Grove, Clonsilla, Dublin, 15 Ireland; 3 School of Medicine, Ahfad University for Women, Omdurman, Sudan

**Keywords:** Injury, Memory decay, Memory recall, Underreporting, Retrospective study, Rate estimation, Effect modification

## Abstract

**Background:**

Injuries represent an important cause of morbidity and mortality worldwide. In retrospective epidemiological studies, estimated rates of reported injuries often decline considerably when information is included from periods more than a few months before the data collection. Such low rates are usually regarded as a consequence of memory decay. It is largely unknown whether the extent of memory decay depends on external factors otherwise affecting injury rates.

**Methods:**

A statistical model was introduced to separate the influence of external factors on true injury rates from effects on memory decay. The relationship between apparent rates and time elapsed between injury occurrence and data collection was described by a parametric regression model. Relationships between memory decay and external factors were modelled by effect modification of the relationship with time. The procedure was applied to data collected in a retrospective household survey, carried out in Khartoum State in 2010, which elicited information about injuries that had occurred during the last year. The survey included 5661 individuals in 973 households, reporting a total of 481 non-fatal injuries.

**Results:**

In the data from Khartoum State, differences in memory recall were observed between socioeconomic groups, with considerably faster memory decay in the lower socioeconomic tertile. In this tertile the estimated probability that an injury which occurred 6 months ago was reported was only 18%, compared to probabilities of about 35% in the remainder of the population. In the lower socioeconomic tertile, in contrast to other groups, a simple exponential model was not sufficient for describing memory decay. Memory decay did not depend on sex, age, urban/rural status or education. Road traffic injuries were subject to less memory decay than injuries due to falls, mechanical causes and burns. Memory decay seriously affected crude overall injury rates and also to some degree estimated relative rates.

**Conclusion:**

In the statistical analysis of retrospective injury data it is important to take into account the effects of memory decay.

## Background

Injuries constitute an important cause of morbidity and mortality worldwide, in developing as well as developed countries, and in 2013 injuries accounted for 10% of the global burden of disease [1]. Although injury rates appear to decline overall, patterns vary widely depending on age, sex, region and time [1]. To explore associations with underlying causative factors it is essential that injury rates are quantified in different populations. Reliable risk estimates are also needed to develop suitable preventive measures and to establish efficient procedures for handling injuries in health care systems.

In large parts of the world it is difficult to carry out prospective studies of injury incidence in well-defined populations. Epidemiological investigations of injuries must frequently rely on data collected retrospectively in surveys dealing with incidents in particular time intervals in the past. It is well-known that memory decay affects data collected in this manner when the time intervals extend over more than a few months [2–7]. It is largely unknown, however, how memory recall depends on the actual time span between data collection and the relevant period in the past. To a major extent it is also uncertain whether memory decay differs essentially between populations or between groups defined by demographic and social factors [5–7].

Most retrospective studies of memory decay have compared apparent injury rates found by subdividing the range for the time between data collection and injury into a certain number of intervals. In several cases rather few and wide intervals have been compared [2, 7, 8]. This may have been necessary because of the structure of the available data, but in principle such procedures represent suboptimal use of information. An alternative approach is to describe memory recall considering a specific mathematical model of the relationship with time between injury and data collection [3, 9–11]. Such models can be fitted to the data by general regression techniques to obtain a more detailed description of the memory decay process. Until now, however, these techniques have not incorporated an assessment of the relationships with other factors affecting injury rates.

The present study will explore these issues by modelling the magnitude of memory decay as a function of the amount of time before information is collected, considering retrospective data from a household survey of injuries carried out in Khartoum State, Sudan. The primary objective is to demonstrate how a relatively simple mathematical relationship between memory decay and time can be established by standard epidemiological procedures. The purpose is also to show how such techniques can be used to investigate whether the relationship depends on demographic and social factors or on injury cause. Finally, implications for the overall estimation of injury rates are considered. The data set has previously been used to explore associations between injury rates and potential risk factors in a Sudanese context [12], to study socioeconomic implications of injuries [13] and to examine use of health services by injured people [14].

## Methods

### Sampling

Injury data for a whole year were collected retrospectively in a household survey conducted in Khartoum State from October to November 2010 [12]. Households were selected by a stratified two-stage sampling procedure, using the most recent sampling frame supplied by the Central Bureau of Statistics (CBS). The selection was carried out separately in an urban stratum, with a population of 4.2 million, and a rural stratum, with a population of 1.0 million. At the first sampling stage, 40 popular administrative units (PAUs) were selected in the urban stratum and 10 in the rural stratum, among a total of 864 and 632 PAUs, respectively. Each PAU was assigned a selection probability proportionate to size. During the next sampling stage, 20 households were found by systematic random sampling within each PAU selected. The household response rate was 97% in the urban and 98% in the rural stratum [12], with information available from a total of 973 households.

### Data collection

Information was collected from each household in an interview performed by a specially trained data collector [12]. Female heads of household were identified as main respondents. In Sudan, female heads of household are considered more knowledgeable of events influencing the family, and national surveys usually rely on them as main respondents [14]. If the female head of household was not present, the next eligible adult was interviewed. If, according to the main respondent, injuries had occurred in the household during the last year, each injured individual was also interviewed about particulars of the event. If an injured individual was absent or less than 18 years old, an adult proxy was assigned. Nobody under the age of 18 years was interviewed alone [14].

A particular questionnaire, developed according to the World Health Organization (WHO) guidelines for surveys on injuries and violence [15], was used to elicit details about each injury reported. The general WHO definition of an injury was briefly explained to the respondents. Any injury experienced was recorded, irrespective of medical care given. Few fatal injuries were reported [13] and the present study deals with the 481 non-fatal injuries reported among the 5661 individuals included.

It was emphasized at the interview that non-fatal injuries should be registered only if they had occurred during the last 12 months before the interview date, and injury dates were recorded.

### Basic handling of follow-up

Only the most recent injury, if any had occurred, was considered for each person at the interview. Thus a retrospective follow-up was introduced, taking as its origin the time of interview and extending the period backwards in time for each person [16], until an injury had occurred or until a complete 12 month period without injuries had been covered.

The true occurrence of injuries was assumed to follow a Poisson process [17] with a uniform injury rate for each person over the year considered. However, the probability that an injury should be reported at the interview was allowed to depend on the length of time between the injury date and the interview [18]. Under these assumptions the occurrence of injuries reported for each person will follow a nonhomogeneous Poisson process, with the rate at any moment given by the product of the actual injury rate and the reporting probability [17].

In the data analysis, the complete year covered before the interview was divided into 11 periods of length 30 days, moving backwards from the interview date, with an additional period of length 35 days at the beginning of the year. As an approximation, reporting probabilities were regarded as constant within each such time period. For simplicity, these periods are referred to as months 1,2,3,...,12 before the interview. The total amount of follow-up time in each such monthly period can be computed for every combination of risk factors considered in each household, with the corresponding number of injuries. It follows from the description above that these numbers can be regarded approximately as independent Poisson distributed variables [17]. The overall follow-up time represented 5365 person-years. Crude injury incidence rates were computed for each monthly interval before the interview, taking into account the total number of person-years experienced in that interval.

### Statistical analysis

In the main statistical analyses, the injury rate of any individual was allowed to depend on the levels of the potential risk factors sex, age and education associated with that particular person. Age was considered in the three categories 0–15 years, 16–44 years and ≥ 45 years. The four categories of education represented no education, khalwa/primary, secondary and diploma/university/postgraduate [12]. Injury rates were also allowed to depend on the factors urban/rural location and socioeconomic status characterizing each household considered. Socioeconomic status was based on a composite household wealth index taking into account home ownership, dwelling type, number of rooms, water source, toilet facilities, lighting, fuel used for cooking and assets owned [12]. The categories considered in the present study corresponded to tertiles of the wealth index.

Statistical analyses of associations with risk factors and time before interview were carried out by Poisson regression with a log-link, including the logarithm of the relevant follow-up time as an offset [19]. Such models with piecewise constant rates are also valid under more general assumptions than those corresponding to a Poisson distribution [19]. The data analysis was performed using PROC GLIMMIX in SAS, version 9.4 (SAS Institute Inc., Cary, NC, USA), adapted to generalized linear mixed models (GLMMs). Random effects were integrated over by Gauss-Hermite quadrature. It was evident that the rates of reported injuries in month 12 before the interview were markedly higher than rates in months 6–11, and thus separate statistical models were introduced to describe memory decay for months 2–11 and month 12, compared to rates in the first month before the interview.

In the basic statistical model considered, the rate of reported injuries in any month *m* = 1, 2, …, 11 was expressed as

1 $$ \lambda \left(\mathbf{x};t\right)={\lambda}_0\left(\mathbf{x}\right)\exp \left({\beta}_1t+{\beta}_2{t}^2+{\beta}_3{t}^3\right) $$

where *t* = *m* − 1. Here **x** denotes a vector of factors potentially influencing injury rates. For *t* = 0 the term *λ*_0_(**x**) represents the injury rate in month 1 just before the interview. The dependence of this term on particular risk factors was modelled in the ordinary manner in epidemiology in a multiplicative fashion including categorical main effects only [20]. This model also incorporated a random effect representing the additional influence of each particular household. The exponential term in (1) involving a cubic polynomial in *t* was used to assess the effect of memory decay, measured relative to the month just before the interview. If it is assumed that all injuries occurring in this month were reported, the exponential term may be interpreted as the probability that an injury is reported in a particular earlier month. When this model is fitted to a specific data set, however, there is no guarantee that the exponential term in (1) will be a decreasing function of the argument *t*, in which case this interpretation may not be suitable.

Additional more complex models with coefficients *β*_1_, *β*_2_, *β*_3_ depending on any of the categorical risk factors considered, corresponding to models with effect modification (or interaction), made it possible to test for the potential influence of such factors on memory decay. For the purpose of data exploration, an alternative model to (1) was also considered with a categorical effect of month before interview, not postulating any particular mathematical relationship between memory decay and time. Month 1 was then regarded as the reference category. For a separate comparison of injury rates in month 12 with those in month 1 only, an analogous categorical model was used.

Models with different specifications of fixed effects were compared using the Akaike information criterion (AIC) [21], defined as twice the difference between the number of parameters included and the log-likelihood. Lower values of AIC represent better descriptions of the data set, as a compromise between simple models with few parameters and more complex models providing a closer fit to the data. Nested statistical models were also compared by chi-square statistics based on values of the log-likelihood.

Information on the major underlying cause of each injury was available for injuries leading to at least one day’s disability. In analyses of memory decay for particular large groups of causes reported, all injuries associated with other causes were regarded as censoring events. Thus accumulation of the backward follow-time was terminated for each person at the time of an injury due to other causes than the one under study.

### Estimation of actual absolute and relative injury rates

For illustrative purposes, unbiased crude estimates of injury rates were computed on the assumption that observations in the first month before the interview were not subject to memory decay. Similar estimates, presumably involving a certain amount of negative bias, were found considering longer cumulative periods before the interview. Crude relative rates were estimated restricting the computation to the relevant categories. As an alternative approach, the absolute injury rate in month 1 was estimated considering predicted values obtained in models specified by Eq. (1). In these models no random household effect was included, as such effects tended to reduce systematically the magnitude of the predicted rates. The predicted values representing different combinations of risk factors were weighted by the corresponding number of person-years in month 1. These analyses also produced model-based estimates of relative rates.

## Results

### Crude rates of reported injuries

The crude incidence rates declined considerably going back 2–5 months before the interview (Table 1). No clear pattern emerged comparing the relatively low rates of reported injuries in months 6–11. For month 12, however, the substantially increased rate of reported injuries was of the same magnitude as the rates for months 3–4.

**Table 1:** Injuries reported and crude incidence rate, by month before interview

Month before interview	Number of injuries reported			Crude incidence (per 100 person-years)	
	Women	Men	Total	Rate	95% CI
1	44	60	104	22.6	18.7–27.4
2	32	49	81	17.9	14.4–22.2
3	20	34	54	12.1	9.2–15.8
4	17	28	45	10.1	7.6–13.6
5	9	10	19	4.3	2.8–6.8
6	8	20	28	6.4	4.4–9.2
7	10	17	27	6.2	4.2–9.0
8	6	11	17	3.9	2.4–6.3
9	6	17	23	5.3	3.5–8.0
10	1	8	9	2.1	1.1–4.0
11	7	9	16	3.7	2.3–6.1
12	25	33	58	11.3	8.7–14.6
Total	185	296	481	9.0	8.2–9.8

### Functional form of the relationship with time

The data set from months 1–11 was analysed considering different Poisson regression models describing the relationship between rates of reported injures and time between injury and interview (Table 2). All models incorporated adjustment for main effects of sex, age, education and urban/rural and socioeeconomic status. Comparing the model with a categorical effect of month before interview with the model without any effect of month at all, the likelihood ratio statistic χ^2^ = 192.23 with 10 degrees of freedom (DF) strongly indicated that rates of reported injuries differed between months overall.

**Table 2:** Alternative Poisson regression models for the relationship with time since injury, involving main effects only^a^

Relationship with time since injury	Number of parameters in model	Akaike information criterion (AIC)	Likelihood ratio χ^2^ compared with categorical model	Degrees of freedom (DF)	Estimated probability that an injury is still reported		Estimated time when reporting probability is 0.5 (months)^b^
					In month 6	In month 11	
Categorical	21	5669.34			0.29	0.17	
Log-cubic^c^	14	5669.39	14.05	7	0.26	0.15	2.1
Log-quadratic^d^	13	5667.43	14.09	8	0.26	0.15	2.2
Log-linear	12	5672.67	21.33	9	0.34	0.12	3.2
No effect of months	11	5841.57	192.23	10	1.00	1.00	

^a^Data included for reported injuries in months 1–11 before interview. All models incorporate main effects of sex, age, urban/rural status, education and socioeconomic status, in addition to a random household effect

^b^Calculated moving backwards in time, starting at the middle of the month preceding the interview

^c^Also includes quadratic and linear terms

^d^Also includes linear term

**Fig. 1:**
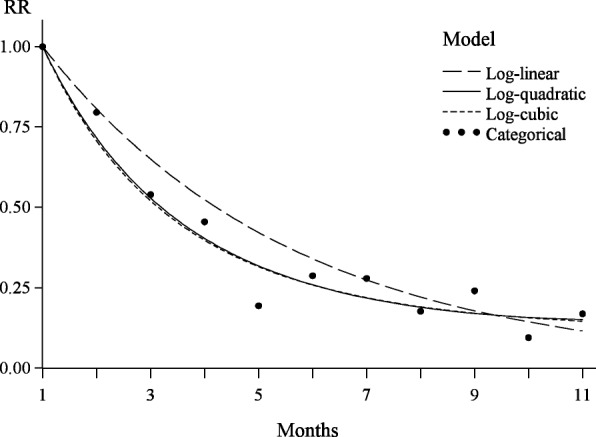
Predicted rates of reported injuries by time since injury, in models involving main effects only. Values along the y-axis (RR) indicate rates relative to month 1 before interview. Curves represent models with a continuous effect of time since injury and dots the model with a categorical effect

On a background model with a categorical effect, postulating no particular form of the relationship with time since injury, the model involving a log-linear effect of month was not supported (χ^2^ = 21.33 with DF = 9), but the model with a log-quadratic effect was (χ^2^ = 14.09 with DF = 8). The log-quadratic model predicted a considerably faster decay in memory during the first 6 months than the log-linear one, with lower recall probabilities at fixed points in time and shorter time until only 50% of the injuries were recalled (Fig. 1 and Table 2). However, predicted rates did not differ substantially in months 8–11 (Fig. 1). Hardly any support was found for a more complex log-cubic rather than a log-quadratic model, and predicted rates were almost identical (Fig. 1). The log-quadratic model also gave the lowest AIC value, 5667.43 (Table 2), with a difference of nearly 2.0 to the second best model, the categorical one (AIC = 5669.34). In all analyses the estimated random household effect was represented by a positive contribution of moderate size. It is concluded that the overall log-quadratic model provides a reasonable parsimonious description of the relationship between rates of reported injuries and the month before interview.

### Dependence of the relationship with time on other factors

Separate analyses were performed on the basis of a log-quadratic relationship with month before interview including effect modification by the potential risk factors sex, age, urban/rural status, education and socioeconomic status (Table 3). Likelihood ratio statistics gave definite support to an effect modification by socioeconomic status (χ^2^ = 10.20 with DF = 4), but no clear support to any other effect modification considered. Among all models with a log-quadratic effect of time since injury, the model with effect modification by socioeconomic status was also the one with the lowest AIC value, 5665.23, considerably below the value found for the log-quadratic model without any effect modification. In contrast, using a model with a log-linear effect of time since interview, little support was found in favour of an effect modification by socioeconomic status (Table 3). The effect modification was also evident in the log-cubic model, but the relatively high AIC values again suggested that a simpler log-quadratic description of the relationship with time since injury was justified.

**Table 3:** Alternative Poisson regression models for the relationship with time since injury, involving effect modifications^a^

Relationship with time since injury	Factor included in effect modification of time since injury	Number of parameters in model	Akaike information criterion (AIC)	Likelihood ratio χ^2^ for effect modification	Degrees of freedom (DF)
Log-quadratic	Sex	15	5668.64	2.79	2
Log-quadratic	Age	17	5674.47	0.96	4
Log-quadratic	Urban/rural status	15	5668.55	2.88	2
Log-quadratic	Education	19	5672.47	6.96	6
Log-quadratic	Socioeconomic status	17	5665.23	10.20	4
Log-cubic	Socioeconomic status	20	5670.66	10.73	6
Log-linear	Socioeconomic status	14	5675.88	0.79	2
Log-quadratic in lower socioeconomic tertile, log-linear otherwise	Socioeconomic status	15	5661.39		

^a^Data included for reported injuries in months 1–11 before interview. All models incorporate main effects of sex, age, urban/rural status, education and socioeconomic status, in addition to a random household effect

**Fig. 2:**
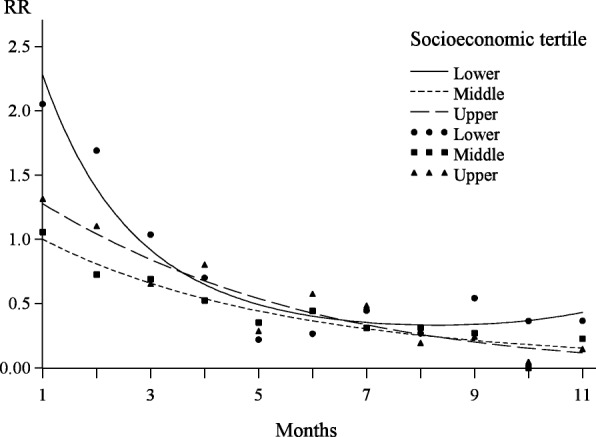
Predicted rates of reported injuries by time since injury in the log-quadratic model involving effect modification by socioeconomic tertile. Values along the y-axis (RR) represent rates, also incorporating the main effect of socioeconomic status, relative to month 1 in the middle socioeconomic tertile. Curves represent the models with a continuous log-quadratic effect and separate points represent the corresponding models with a categorical effect

Considering the relationship with time since injury within tertiles of socioeconomic status, the category with the lower status exhibited a rather different behaviour from the other two (Fig. 2 and Table 4). Memory decay initially occurred at a much faster rate in this group, with substantially lower probabilities that an injury should be reported during the first 6 months. A log-quadratic function was clearly needed within this tertile to provide a reasonable description of the relationship with time since injury. Taking into account the standard errors (SE) associated with the estimates of the quadratic coefficients in the middle and upper socioeconomic tertiles (Table 4), no justification of any quadratic term was essentially found in these groups. The injury rate in month 1 was also much higher in the lower socioeconomic tertile, twice the value found in the middle tertile (Table 4). Despite these major differences between socioeconomic groups, the estimated rates of reported injuries did not differ substantially after about 7 months (Fig. 2).

**Table 4:** Log-quadratic relationship with time since injury, within tertiles of socioeconomic status^a^

Tertile of socioeconomic status	Crude injury rate in month 1 before interview (per 100 person-years) (SE)	Regression coefficient of linear term (per month) (SE)	Regression coefficient of quadratic term (per month) (SE)	Estimated probability that an injury is still reported		Estimated time when reporting probability is 0.5 (months)^b^
				In month 6	In month 11	
Lower	32.3 (4.6)	−0.527 (0.080)	0.036 (0.009)	0.18	0.19	1.5
Middle	16.1 (3.2)	−0.215 (0.104)	0.003 (0.012)	0.37	0.15	3.4
Upper	19.3 (3.6)	−0.200 (0.101)	−0.004 (0.012)	0.33	0.09	3.3

^a^Data are included for reported injuries in months 1–11 before interview. The models incorporate common main effects of sex, age, urban/rural status and education, in addition to a random household effect

^b^Calculated moving backwards in time, starting at the middle of the month preceding the interview

A separate analysis allowing for a log-quadratic relationship in the lower socioeconomic tertile only, otherwise considering separate log-linear relationships, produced an AIC value of 5661.39 (Table 3), substantially below the values achieved in other models. Thus this model had the strongest support in the data set.

### Relationship with time for separate injury causes

**Fig. 3:**
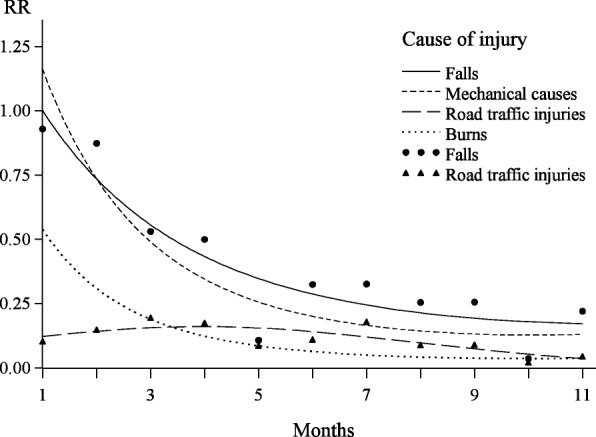
Predicted rates of reported injuries by time since injury in log-quadratic models, according to cause of injury. Values along the y-axis (RR) represent rates relative to month 1 for falls. Curves represent the models with a continuous log-quadratic effect and separate points represent the corresponding models with a categorical effect. For simplicity categorical values are shown for falls and road traffic injuries only

Among the 378 injuries reported from months 1–11, leading to at least one day of disability, a total of 123 injuries were ascribed to falls, 111 to mechanical causes, including cuts, stabs, struck by object etc., 57 to road traffic crashes and 43 to burns. The remaining 44 cases represented various other minor categories. Separate log-quadratic Poisson regression analyses of the rate of reported injuries with regard to time since injury were performed for the four specific groups of causes, including data from months 1–11. To avoid problems associated with overadjustment in small subgroups, these analyses were only adjusted for the household random factor. Falls and mechanical causes showed rather similar relationships with time since injury (Fig. 3), although memory decay seemed to be slightly stronger at short time intervals for mechanical causes than falls. Burns showed an even stronger tendency to early memory loss, with a particularly low estimated rate of reported injuries at the end of the 11 month interval. The estimated curve of the time dependency for road traffic incidents exhibited a very different pattern, in that rates did not seem to decline during the first 6 months. In fact a weak tendency to higher rates late in this period was suggested (Fig. 3). Subsequently, the estimated rate of reported road traffic injuries dropped slowly.

### Comparison of rates in month 12 with month 1

Particular analyses restricted to follow-up in months 1 and 12 made it possible to compare rates of reported injuries a short time before the interview with those presumably reflecting the situation nearly a year ago. Overall, with adjustment for the same potential risk factors as in the analyses of months 1–11, the injury rate in month 12 vs. that in month 1 was 0.52 (95% confidence interval (CI) 0.38–0.71). No definite support was found for any effect modification of the association with time before interview by other factors, with the strongest suggestion seen for age groups (χ^2^ = 3.90 with DF = 2). In this case the rates in months 12 vs. those in month 1 were 0.39, 0.51 and 1.02, respectively, for the age groups 0–15 years, 16–44 years and ≥ 45 years. There was no indication at all that rates in month 12 vs. those in month 1 differed between socioeconomic tertiles (χ^2^ = 0.02 with DF = 2). The estimated relative rates were 0.50, 0.54 and 0.53, respectively, for the lower, middle and upper tertiles.

### Overall injury rate estimates

Crude injury rates were computed considering cumulative periods of different length before the interview (Table 5). The crude injury rate dropped from 22.6 injuries per 100 person-years, taking into account data from month 1 only, to 8.7 injuries per 100 person-years when the entire 11 month period was included (Table 5), representing a 62% decline. The estimate was only slightly increased when the additional month 12 was also taken into account. Corresponding model-based estimates found for the same time intervals, not including any effect of time, were very similar (Table 5). Estimates based on models with a log-quadratic effect of time before interview were slightly higher than the crude 1 month estimate (23.3 vs. 22.6 injuries per 100 person-years; Table 5).

**Table 5:** Overall injury rate and relative rates among socioeconomic tertiles estimated by different procedures^a^

Estimation procedure	Months included in recall period	Model effect of time since injury	Total rate estimate (per 100 person-years)	Relative rate, lower vs. middle socioeconomic tertile	Relative rate, upper vs. middle socioeconomic tertile
Crude^b^	1	None	22.6	2.01	1.20
Crude^b^	1–3	None	17.6	1.99	1.20
Crude^b^	1–11	None	8.7	1.68	1.15
Crude^b^	1–12	None	9.0	1.69	1.16
Predicted model-based value^c^	1	None	22.6	1.62	1.48
Predicted model-based value^c^	1–11	None	8.8	1.62	1.19
Predicted model-based value^c^	1–11	Common log-quadratic effect	23.3	1.61	1.19
Predicted model-based value^c^	1–11	Separate log-quadratic effects within socioeconomic tertiles	23.3	2.27	1.28

^a^Estimates are computed for various cumulative periods among months 1–12 before interview

^b^Based directly on number of injuries and person-years

^c^Incorporates adjustment for main effects of sex, age, urban/rural status and education

### Overall relative rate estimates

The relative rate of reported injuries for the lower socioeconomic tertile vs. the middle one showed a moderate decrease from 2.01 to 1.68 when crude values were compared for month 1 only and for the full 1–11 month cumulative period (Table 5). Among model-based estimates, the corresponding relative rates were all rather similar, approximately 1.6, using models without a time effect or with a common log-quadratic effect. However, in the model with separate log-quadratic effects of time in the socioeconomic tertiles, the estimated relative rate was noticeably higher (Table 5). The overall support for retaining the main effect of socioeconomic status in this model was very strong (χ^2^ = 16.19 with DF = 2). This was also the case for the main effect of sex (χ^2^ = 25.91 with DF = 1), with an estimated relative rate of 1.69 for men vs. women. In contrast, no definite support was found for non-zero main effects of age, urban/rural status or education. In general, model-based estimates of relative rates for sex, age, urban/rural status and education were quite similar, regardless of whether time since injury was included as a risk factor in the model or not.

## Discussion

This paper has shown how a simple parametric statistical model may be used for assessing the effect of relevant factors on memory loss, depending on the time since an injury occurred. In the data from Khartoum State a basic exponential model, corresponding to a constant rate of memory loss [22], did not suffice for describing the overall relationship. Both in the complete data set and in the particular lower socioeconomic tertile, a model involving an exponential function with a quadratic expression in time was needed, although basic exponential models were still adequate in the other tertiles. The general model was also used for exploring memory decay after injuries due to specific causes, suggesting that relationships with time may differ, with a slower memory decay after road traffic injuries.

In some studies of memory decay after injuries, another external data source has been available, providing information about all or nearly all injuries that could potentially be reported [2, 8, 23–25]. The statistical analysis can then proceed in a different manner, with direct estimation of reporting probabilities. In studies based on comparison of rates at different times, various mathematical expressions have been introduced to model the relationship between memory decay and time since injury in particular groups [3, 9–11, 26]. These models correspond to the last part of our formulation (1), representing the contribution to the rate of reported injuries made by the time variable. The fundamental assumption that injuries reported from the period immediately before data collection reflect the true state was formulated explicitly by Massey and Gonzalez already in 1976 [3], and this assumption is underlying the arguments used in various subsequent papers on memory recall [4, 9]. In some studies the mathematical relationship has been fitted separately to subsets of the data representing particular injury categories [3, 9, 11, 27]. Yet, to our knowledge, no one has previously considered a joint statistical model for the rate of observed injuries, combining one term representing the true injury rate in the first time period depending on overall risk factors and a second term describing memory decay moving further back in time.

Although Eq. (1) has a very simple structure, the interpretation of the first term *λ*_0_(**x**) as the true injury rate requires that the exponential second term is equal to unity for time *m* = 1 , i.e. *t* = 0. Which factors should be included in modelling the first term depends on what can realistically be assumed to influence true injury rates. It seems reasonable to investigate whether the coefficients *β*_*j*_ in the second term depend on the same factors, although these coefficients have a completely different interpretation, relating to memory decay rather than true injury incidence. Thus the analysis of a particular data set may lead to the conclusion that a certain factor affects memory decay but not true injury rates. The situation is rather unusual in that non-hierarchical statistical models, involving effect modification (or interaction) relating to a particular factor but not main effects of the same factor, may be relevant. In this connection it is essential that main effects are defined with respect to time *t* = 0, considering true injury rates, but these are the quantities of primary interest anyhow.

The functional form of mathematical relationships considered in work on memory recall after injuries has largely been inspired by the models introduced by Massey and Gonzalez [3] for studying overall injury rates in USA on the basis of national surveys. Let *μ*(*t*) be the true rate of reported injuries at time *t* before the data collection, assuming for the moment that dependence on other risk factors is not explicitly taken into account. Moreover, let

2 $$ \overline{\mu}(t)=\frac{1}{t}{\int}_0^t\mu (w) dw $$

be the true average rate of reported injuries for the entire time interval of length *t*, moving back in time from the data collection. Massey and Gonzalez [3] considered three alternative models describing the relationship between time and the average rate:

3 $$ \overline{\mu}(t)=\alpha \exp \left(-\beta {t}^2\right), $$

4 $$ \overline{\mu}(t)=\alpha \exp \left(-\beta t\right), $$

5 $$ \overline{\mu}(t)=\alpha +\beta t+\gamma {t}^2 $$

Similar relationships were considered in a more recent study of overall injury rates in USA [11], supplemented by linear and cubic analogues to the quadratic relationship in Eq. (5). In a study of occupational injuries in USA [10], the rate *μ*(*t*) itself was assumed to follow a relationship with time given by the right hand side of one of the Eqs. (3), (4) and (5) (or a simple linear analogue to (5)). Finally, a linear relationship was considered in another study of occupational injuries in USA [9].

A clear distinction has not always been made in the literature between the rate *μ*(*t*) at any moment and the average rate $\overline{\mu}(t)$ for the whole time interval of length *t*. The focus in some papers [3, 11] on the average rate appears quite reasonable when the main purpose is to compare the bias introduced by underreporting of injuries considering data from cumulative intervals of different length. This has the disadvantage, however, that corresponding crude estimates of the average rate from various overlapping intervals are not independent [3]. As in other epidemiological models dealing with changes over time, it seems more natural to formulate the basic mathematical relationships in terms of the rate itself. Furthermore, adoption of expressions such as those appearing on the right hand sides of Eqs. (3) or (4) for either *μ*(*t*) or $\overline{\mu}(t)$ is not consistent with similar expressions being valid for the other quantity, as may be seen from Eq. (2). If a polynomial is used as on the right hand side of Eq. (5), the corresponding expression for the other quantity is still a polynomial of the same degree, but the relative magnitude of the coefficients is different.

Replacing the average rate on the left hand sides of Eqs. (3) and (4) with the rate *μ*(*t*), the right hand sides constitute particular cases of the corresponding relationship with time given by our formulation (1). Expression (4) then represents standard exponential memory decay, but if a more complex model is needed, it seems natural to consider general polynomials in the exponent as in (1), not just a single second degree term in time as in (3). The expression on the right hand side of (3) imposes the condition on the relationship with time that the first derivative vanishes when *t* = 0 and the second derivative is negative for small time values, properties which cannot be taken for granted. Pure polynomial models as given by the right hand side of Eq. (5) have the general disadvantage that they may lead to predicted negative values of the injury rates. With relatively little memory decay over the time span considered [9], this is not necessarily a major problem, but in a study such as ours it would be.

In the present data set, the rate of reported injuries declined substantially moving back more than a few months from the time of interview. It appears that the great majority of the injuries occurring at least 5 months ago were not reported. This is consistent with recall studies of injuries carried out in other African countries as Ghana [5] and Tanzania [6] or in the overall Sudanese population [7]. However, the particular problems relating to injuries reported to occur almost a year before data collection appear to be considerably more pronounced in the current study. A weak tendency to higher rates for recall periods approaching 12 months was seen in Tanzania [6] but not in Ghana [5]. Although some degree of general imprecise specification of injury dates may have affected our observations, the high rate of reported injuries in month 12 seems to require a different explanation. It is likely that forward telescoping, the tendency to report incidents as if they had occurred more recently than they actually did [28], may have affected injuries occurring before the 12 month period covered here. If this is correct, a major part of the injuries assigned to month 12 do not really belong to the time range of our study. The observation that the apparent elevated rates in month 12 were not strongly associated with other factors lends some support to this idea.

This study found no major differences in memory decay between demographic groups except among socioeconomic categories. Few studies have shown definite differences of this kind. With a classification indicating whether injuries were reported to occur earlier than 4 weeks before data collection or not, the study at the national level in Sudan [7] found less memory decay with increasing age. In the study from Tanzania [6] more memory loss over time was found in rural areas, but no essential difference was seen between urban areas in the narrow sense and periurban areas, a comparison more similar to the one carried out in the present study. No major differences were found in Ghana [5]. In recall studies in the general population of USA [3] and in occupational studies [9, 10] faster memory decay has been indicated for certain groups of young people.

The recall data from the lower socioeconomic tertile in this study gave quite a different impression from data in the middle and upper tertiles. It is reasonable to consider the faster memory decay in the lower tertile in connection with the higher rate of injuries reported in this group in month 1. It is certainly possible that this group experienced more memory loss in the first few months after an injury and that the true injury rate was elevated. A simple explanation could be that low socioeconomic status was associated with more instability in society, affecting both true injury risk and survey response. Another possibility is that members of this group had different ideas about incidents that should be reported. If some respondents tended to include more very minor recent incidents, such cases might be more easily ignored after a couple of months. A third possibility is that forward telescoping was a more serious problem for short time intervals in this category. No major contrast in recall was found between groups defined by a wealth index in the national study in Sudan [7].

Comparing separate causes, the national Sudanese study [7] found least memory decay for injuries caused by falls. This is consistent with the relatively slow memory decay seen for falls in the present study. However, most differences in memory decay according to causes were not very large in the national study, and road traffic crashes did not display an obviously different pattern from other causes. In our study, memories of recent road traffic injuries may have been subject to backward telescoping [28], leading to relatively few injuries being reported very close to data collection. Most other studies did not consider memory decay according to causes in a similar manner, although one national study in USA [3] found the slowest rate of memory decay for injuries caused by moving motor vehicles, with falls also exhibiting relatively slow decay. Several studies [4–6, 9] have shown much slower memory decay for serious injuries. In the present study, classification according to severity was based on days of normal activity lost, and for many injuries this information was not yet available at data collection, so a similar classification according to recall was not feasible.

The decline in crude rates with longer recall periods in our study clearly illustrates the problem of using overall estimates based on values collected over long periods. A 3 month period is often considered reasonable for obtaining fairly reliable estimates [4–6] but even in that case our crude estimate was 22% lower than the estimate for a 1 month period. On the other hand, sampling errors increase with very short recall periods, and potential forward telescoping in intervals close to data collection may be another reason why extremely short recall periods should be avoided. To compensate for the sampling error associated with estimates from a short period before data collection only, overall rate estimates have in some recall studies [9, 10, 26] been based on predicted values from a regression model. This value is computed at the lower end of the relevant range, representing a time value close to data collection. Such procedures are likely to introduce more stability in estimates, in particular if memory decay is relatively modest. In our data, the predicted overall rates found by this procedure were of the same magnitude as the crude estimates.

However, the relative rates among socioeconomic tertiles were affected by the adjustment carried out in the model-based approach. Use of separate quadratic relationships in the distinct socioeconomic groups led to a larger relative risk estimate for the lower vs. the middle tertile, testifying that use of the correct model with effect modification by socioeconomic status may be important. For factors not influencing memory decay to any appreciable extent, our model-based relative rate estimates were rather similar to those found in previous analyses of the same data in the large urban stratum [12], ignoring memory decay. Other studies investigating the effect on relative rate estimates of taking memory decay into account [9, 10] also found a considerably smaller change in relative than in absolute rates.

In a survey-based study such as the present one, the response from any particular participant may be subject to errors of several kinds [28]. Some injuries may not be reported for various reasons, some incidents reported may not have occurred, and the injury time recorded may be biased in either direction. The data set considered in this study, with its background from Khartoum State, offers unique possibilities, but it is not extremely large, especially when attention is confined to population subgroups. Thus it was not practicable to carry out complete analyses of memory decay for separate causes within socioeconomic categories. In interpreting the decline in apparent rates as an expression of memory decay, the important assumption is made that true underlying injury rates are nearly uniform over time. This seems reasonable when the environment is not subject to major seasonal fluctuations.

## Conclusions

Ordinary techniques in epidemiology for modelling effects of risk factors on incidence rates can be adapted to retrospective studies to separate the effects on true injury rates from those influencing reporting probabilities. Briefly, the former should be represented by main effects in the statistical model and the latter by effect modification of terms representing the effect of time since injury. For a data set of moderate size, such as the one from Khartoum State studied here, it may be advisable to use a relatively simple regression model of the time effect to capture most of the information available. With substantially larger data sets, more descriptive semi-parametric alternatives can be relevant. Thus splines, representing combinations of different polynomials in adjacent intervals with smooth transitions, have been used to describe memory decay after fires [26]. The basic idea behind our approach, modelling effects on memory decay by allowing the coefficients describing the time effect to depend on other factors, should still be useful. Memory decay can affect many results in epidemiological studies, generating recall bias [29], and it is important to evaluate its magnitude also in retrospective injury studies.

## Abbreviations

AIC: Akaike information criterion
CBS: Central Bureau of Statistics
CI: Confidence interval
DF: Degrees of freedom
GLMM: Generalized linear mixed model
PAU: Popular administrative unit
RR: Relative rate
SE: Standard error
WHO: World Health Organization
